# The Relationship between Occupational Exposure to Lead and Hearing Loss in a Cross-Sectional Survey of Iranian Workers

**DOI:** 10.3389/fpubh.2016.00019

**Published:** 2016-02-16

**Authors:** Masoumeh Ghiasvand, Saber Mohammadi, Brett Roth, Mostafa Ranjbar

**Affiliations:** ^1^Forensic and Toxicology Department, Iran University of Medical Sciences, Tehran, Iran; ^2^Occupational Medicine Department, Iran University of Medical Sciences, Tehran, Iran; ^3^Division of Medical Toxicology, Department of Emergency Medicine, UT Southwestern Medical Center, Dallas, TX, USA; ^4^Medical School, Iran University of Medical Sciences, Tehran, Iran

**Keywords:** blood lead level, hearing loss, lead ototoxicity, pure tone audiometry

## Abstract

**Objectives:**

Ototoxic effect of exposure to lead has been reported by many researchers. This study was undertaken with a view to investigate the relationship between blood lead level (BLL) and hearing loss in workers in a lead-acid battery manufacturing plant in Tehran, Iran.

**Methods:**

In a cross-sectional study, 609 male workers were recruited from different locations in the factory. Association between BLL and hearing loss in different frequencies were measured. Relationships were analyzed by logistic regressions. Statistical significance was defined as *p*-value <0.05.

**Results:**

Six hundred nine male workers with mean age 40 ± 7 years and mean noise exposure level of 80 (75–85) dB were evaluated. BLLs were categorized into four quartiles, and hearing loss in each quartile was compared to the first one. In our regression models, BLL was associated significantly with high frequency hearing loss, adjusted odds ratios for the comparison of the fourth, third, and second quartiles to the first one are respectively: 3.98 (95% CI: 1.63–9.71, *p* < 0.00), 3.05 (95% CI: 1.28–7.26, *p* < 0.01), and 2.89 (95% CI: 1.11–7.51, *p* < 0.03).

**Conclusion:**

This study showed a dose–response relationship between BLL and hearing loss, after adjusting for potential confounders (age, body mass index, work duration, smoking, and occupational noise exposure) in logistic regressions. It is concluded that periodic hearing assessment by pure tone audiometry in workers exposed to lead should be recommended. However, additional studies are required to clarify the mechanisms of lead ototoxicity.

## Introduction

Work-related ototoxic compounds include heavy metals, noise, solvents, and certain drugs, used in industry. They are known for their neurotoxic effects both on central and peripheral nervous systems. Mechanisms of ototoxicity include injury to the sensory cells, peripheral nerve endings of the cochlea and direct cochlear toxicity (1).

Lead (Pb) is a heavy metal common in nature and is a potent occupational poison affecting multiple body systems (2).

Recent findings indicate that inorganic Pb2+ can substitute for Ca2+ with certain intracellular Ca2+-binding proteins. Such observations suggest a variety of hypotheses for understanding the molecular basis of its toxic action, especially in reference to both the acute and chronic low level exposure models of neurotoxicity. Pb2+ interacts with calmodulin with an affinity at least equal to that for Ca2+ (3).

Some studies show the effects of inorganic Pb2+ on immature rat brain mitochondrial respiration. Low Pb2+ concentrations produced an increase in mitochondrial respiration and led to a net decrease of mitochondrial Ca2+ uptake, increased mitochondrial Ca2+ efflux, increased mobilization of Ca2+ from endoplasmic reticulum, and interference with the ATP-driven Ca2+-ATPase located in the plasmalemma (4–7).

Some animal model studies have also demonstrated that lead may have an ototoxic effect (8–12).

Significant auditory nerve toxicity in human subjects has been demonstrated in many epidemiological surveys (2, 13–20).

Except in some under developed and developing countries, there is strong governmental pressure to reduce the use of lead in Industry. Many countries have banned the use of lead-based paints and, despite strong opposition from the industries, they are seriously considering similar ban on lead-acid batteries in the cars (21–26).

However, there are many sources of exposure to lead in Iran: air pollution, water pipes, or leaded paints, occupational lead exposure is an important health issue in Iran and mine workers, employees of paint factories, workers of copying centers, drivers, and tile-making factories are in higher risk of lead toxicity. The various processes involved in lead-acid battery manufacturing and recycling are a significant source of exposure to lead. Iran is among the countries that are vigorously enforcing reduction of lead in the industries (27).

There is a paucity or lack of data regarding the associations between the hearing loss and lead exposure in Iranian workers, to obviate this lacuna, this study was undertaken as a cross-sectional study to evaluate the relationship between blood lead level (BLL) and hearing loss in lead-exposed workers in a lead-acid battery manufacturing factory in Tehran.

## Materials and Methods

A cross-sectional approach was selected for this study, and it was conducted in a lead-acid battery factory located in Tehran. Six hundred nine male workers with mean age 40 ± 7 years were recruited from the factory.

Personal information, such as age, work duration, previous work experiences, disease history, use of medications (aminoglycosides, loop diuretics, non-steroidal anti-inflammatory drugs, or antineoplastic drugs during the past month), smoking, and drinking habits, were obtained through a questionnaire. Exclusion criteria included exposure to ototoxic chemical or drugs, suffering from a systemic disease (thyroid disorders, dyslipidemia, diabetes mellitus, hypertension) that affects hearing, or pre-employment hearing loss. The systemic disease was classified based on self-reported physician diagnosis or current use of relevant drugs.

We excluded 21 subjects who did not participate in audiometric examination.

Blood samples were collected and analyzed for levels of lead with Atomic Absorption Spectrometer of Specter AA 220, USA Varian. In order to determine the relationship between BLL and hearing loss and the magnitude of biological response, BLLs were categorized into four quartiles (the first quartile: BLL < 10 μg/dl, the second quartile: BLL 10–19 μg/dl, the third quartile: BLL 20–39 μg/dl, and the fourth quartile: BLL ≥ 40 μg/dl), and hearing loss in each quartile was compared to the first quartile (28).

Body weight was measured in light indoor clothing and recorded to the nearest kilograms. Height was measured to the nearest centimeter without shoes. Body mass index (BMI) was calculated as weight (kilograms) divided by height squared (meter square), and abnormal BMI was defined as BMI > 25.

Smokers were categorized into two groups, the first group (G1) who smoked <6 pack/year and the second group (G2) who smoked ≥6 pack/year.

The work environment was tested for total respirable lead in fumes and particles. Total respirable lead was as high as 25.3 μg/m^3^ (29).

### Noise Exposure Assessment

A team of occupational hygienists working in the field of health and safety executive (HSE) managed noise monitoring. Digital sound level meter (EXAIR, model 9104: Cincinnati, OH, USA) with 4-digit backlit LCD was placed in 25 various stations according to international standard organization (ISO 1999). In this study, after measuring sound level in different parts of the factory, 5 areas with high levels of noise were omitted; eventually 20 areas of the factory with the noise level of 75–85 dBA were selected as non-noisy areas. The time-weighted average (TWA) was 80 dBA.

### Audiometric Measurement

Audiometry was taken by a qualified audiometrist with a standard audiometer (AD 229e, Interacoustics A/S, Assens, Denmark) after at least 14 h of end of shift in an acoustic chamber, meeting the American National Standards Institute (ANSI S3.1-1991) standards. Pure tone air conduction hearing threshold was obtained for both ears at frequencies of 0.5, 1, 2, 4, 6, and 8 kHz over an intensity range of 0–120 dB.

According to 1996 ANSI audiometric standards, normal hearing range was defined as a pure tone average (PTA) of 25 dB or less. In this study, we defined hearing loss as a PTA > 25 dB in either ear.

Low frequencies are the frequencies ≤2 kHz and high frequencies are the frequencies more than 2 kHz.

Air conduction hearing thresholds in decibels were measured in each ear at 0.5, 1, 2, 4, 6, and 8 kHz. Audiological frequencies were used as biomarkers for ototoxicity in adults with chronic lead exposure. All audiological test data were obtained concurrently with the collection of blood samples from the study participants.

### Statistical Analysis

All statistical analyses were performed with SPSS software (version 16). The χ^2^ and Fisher’s exact test were used for comparison between the qualitative variables, and *T*-test was used for quantitative variables with normal distribution. Odds ratios (ORs) with 95% confidence interval (95% CI) were used for comparing risks. In order to determine the relationship between BLL and hearing loss and the magnitude of biological response, BLLs were categorized into four quartiles (the first quartile: BLL < 10 μg/dl, the second quartile: BLL 10–19 μg/dl, the third quartile: BLL 20–39 μg/dl, and the fourth quartile: BLL ≥ 40 μg/dl), and hearing loss in each quartile was compared to the first quartile. Logistic regression analysis was used to measure the relationship between exposure to lead and hearing loss defined as PTA > 25 dB in one or both ears. Two-sided *p*-value <0.05 was considered for statistical significance.

For ethical clearance, oral informed consent was obtained from all workers before interview, and all of steps were carried out in a quiet place with adequate privacy. All the collected data were kept confidential, and written informed consent was obtained from HSE manager of the factory.

The study protocol was approved by the ethical committee of the Rasoul-e-Akram Hospital of Iran University of Medical Sciences in Tehran, Iran.

## Results

The mean age, mean of work duration, and mean BLL are, respectively, 40 ± 7, 8 ± 4 years, and 37.85 ± 17.55 μg/dl.

Table 1 describes the characteristics of participants in regard to BLLs. For the first and the fourth quartiles, means were, respectively, 38 ± 7 and 40 ± 6 years for age, 6 ± 4 and 8 ± 3 years for work duration, 8 ± 1 and 51 ± 10/μg/dl for BLL, and 22 ± 9 and 29 ± 14 dB for hearing high frequencies. No significant differences were observed in hearing low frequencies between the 4 groups of BLL exposure (the *p*-values were *p* > 0.05).

**Table 1:** **Compare different variables between different groups**.

Variables	Group 1 Mean **±** SD	Group 2 Mean **±** SD	Group 3 Mean **±** SD	Group 4 Mean **±** SD
Age/year	38.36 ± 7.34	38.43 ± 5.87	40.50 ± 6.50	40.58 ± 6.67
Work duration/year	6.50 ± 4.28	8.67 ± 4.19	7.51 ± 4.45	8.76 ± 3.81
Smoking (pack/year)	1.38 ± 0.72	1.12 ± 0.39	1.25 ± 0.59	1.54 ± 0.79
BMI	22.62 ± 2.44	26.52 ± 2.98	26.17 ± 2.50	26.50 ± 3.28
BLL/μg/dl	8.27 ± 0.98	14.87 ± 2.76	30.70 ± 5.26	51.43 ± 10.07
HLFR/dB	19.35 ± 10.22	19.30 ± 8.63	19.96 ± 7.71	19.46 ± 6.89
HLFL/dB	18.81 ± 10.18	19.07 ± 6.83	21.76 ± 32.85	19.95 ± 7.87
HHFR/dB	22.38 ± 9.61	26.90 ± 13.44	27.341 ± 1.99	29.50 ± 14.00
HHFL/dB	24.01 ± 12.51	26.97 ± 12.54	27.93 ± 12.19	29.87 ± 12.69

*BMI, body mass index; BLL, blood lead level; HLFR, hearing low frequencies in right ear; HLFL, hearing low frequencies in left ear; HHFR, hearing high frequencies in right ear; HHFL, hearing high frequencies in left ear*.

We found significant differences for high frequency hearing loss in regard to the four groups of BLL exposure, the number of persons with high frequency hearing loss in the first group was 10 (27%), in the second group was 37 (46%), in the third group was 126 (52%), and in the fourth group was 148 (51%) (*p* < 0.001).

In the Table 2, logistic regression analysis was used to estimate the associations between high frequency hearing loss defined as PTA > 25 dB in one or both ears and BLLs expressed as quartiles.

**Table 2:** **Correlation between high frequency hearing loss and variables of BLL, age, BMI, work duration, and smoking by logistic regression analysis**.

Variables	Adjusted OR	95% CI	*p*-Value
BLL/μg/dl			
G1	–	–	–
G2	2.89	1.11–7.51	0.03
G3	3.05	1.28–7.26	0.01
G4	3.98	1.63–9.71	0.00
Age/year	1.11	1.08–1.15	0.00
Work duration/year	1.01	0.97–1.06	0.43
BMI	0.96	0.91–1.01	0.19
Smoking G1	0.81	0.46–1.41	0.46
Smoking G2	1.23	0.61–2.47	0.55

*BMI, body mass index; BLL, blood lead level; G1, first quartile; G2, second quartile; G3, third quartile; G4, fourth quartile*.

In our regression models, BLL was associated significantly with high frequency hearing loss, adjusted ORs for the comparison of the fourth, third, and second quartiles to the first one are, respectively, 3.98 (95% CI: 1.63–9.71, *p* < 0.00), 3.05 (95% CI: 1.28–7.26, *p* < 0.01), and 2.89 (95% CI: 1.11–7.51, *p* < 0.03). The effect of noise was adjusted, and it was not significant (*p* = 0.21).

After adjusting of potential covariates (age, work duration, BMI, and smoking), results showed a dose–response relationship between BLL and hearing loss.

We did not observe any statistically significant association between smoking, work duration or BMI with hearing loss (Table 2).

## Discussion

This study showed that high frequency hearing loss in the range of 4, 6, and 8 kHz, not only was significantly correlated with BLLs ≥10 μg/dl but also showed a dose–response relationship, *p*-value <0.001.

Our results were consistent with previous studies demonstrating ototoxic effects of lead exposure (2, 13–20, 30).

Recent findings indicate that inorganic Pb2+ can substitute for Ca2+ with certain intracellular Ca2+-binding proteins. Such observations suggest a variety of hypotheses for understanding the molecular basis of its toxic action, especially in reference to both the acute and chronic low level exposure models of neurotoxicity. Pb2+ interacts with calmodulin with an affinity at least equal to that for Ca2+ (3).

In humans, blood lead concentrations correlated significantly with abnormalities in the recorded evoked potentials in several studies. Forst reported a significant relation between current BLLs and elevated hearing thresholds (31). Bleecker reported an association between current mean 28 μg/dl and lifetime weighted average 39 μg/dl BLLs and auditory dysfunction (30). Several other investigations have shown correlations between current mean BLLs of 42–57 μg/dl and auditory dysfunction (13–15, 19). Farahat reported a significant correlation between current BLLs and increased hearing thresholds. Exposed workers (BLL’s mean: 37 μg/dl) had significantly elevated hearing thresholds compared to the controls (20).

Animal studies have provided conflicting results when evaluating the effects of lead on hearing. In guinea pigs, dysfunction of the eighth nerve was induced by high-dose lead exposure but did not induce electrophysiological dysfunction of the organ of Corti and the stria vascularis (10). By contrast, one study in monkeys showed that the auditory-evoked response at levels from the auditory nerve to the cerebral cortex did not significantly differ as a function of lead exposure (9). In another study, three of six monkeys exposed for lifetime to lead, with high current blood lead concentrations showed elevated thresholds for pure tones (11). Other study of long-term exposed monkeys, BLLs of 35 μg/dl did not show significant effects on evoked potentials, at level of 55 μg/dl, these effects were significant (12). BLLs of 35–40 μg/dl in monkeys exposed from birth up to 2 years of age had no significant effects on auditory function (8).

However, two studies have shown opposite findings with no relation to hearing loss and BLLs (32, 33).

Some limitations in this study should be considered. The present study was conducted with a cross-sectional design that may preclude inferences of causality in the association between lead exposure and hearing loss.

## Conclusion

This study showed a dose–response relationship between BLL ≥10 μg/dl and high frequency hearing loss, after adjusting for potential confounders (noise, age, BMI, work duration, and smoking) in logistic regressions. Periodic hearing assessment by pure tone audiometry in workers exposed to low level of lead even if the ambient noise level is <85 dB should be recommended. However, additional studies are required to clarify the mechanisms of lead ototoxicity. Despite laws established in the 1970s to make people aware of the dangers of lead and its poisonous effects, lead poisoning remains a common, yet preventable, environmental health problem in the world. By understanding, identifying, and safely removing sources of lead, we can prevent its devastating and irreversible effects.

Occupational lead exposure is an important health issue in Iran, and we recommend identifying, eliminating or controlling sources, and monitoring environmental exposures and hazards to prevent lead poisoning.

## Author Contributions

MG and MR: participated in the design of the study and performed the data collection. MG and SM: performed the statistical analysis and interpretation of data. SM, MR, and MG: conceived of the study, and participated in its design and coordination. MG: drafted the manuscript. BR and MG: revising it critically for important intellectual content. MG: final approval of the version to be published. All authors read and approved the final manuscript.

## Conflict of Interest Statement

The authors declare that the research was conducted in the absence of any commercial or financial relationships that could be construed as a potential conflict of interest.

## Abbreviations

BLL, blood lead level; BMI, body mass index; PTA, pure tone audiometry.
